# In Situ Synthesis of Crystalline MoS_2_@ZIF-67 Nanocomposite for the Efficient Removal of Methyl Orange Dye from Aqueous Media

**DOI:** 10.3390/mi14081534

**Published:** 2023-07-30

**Authors:** Tahreem Haq Nawz, Muhammad Talha Masood, Amna Safdar, Muhammad Shahid, Tayyaba Noor, Muzammil Hussain, Ayesha Razi, Malik Adeel Umer

**Affiliations:** 1 School of Chemical and Materials Engineering (SCME), National University of Sciences and Technology (NUST), H-12 Sector, Islamabad 44000, Pakistan; tahreem.bs.chemistry@gmail.com (T.H.N.); talha.masood@scme.nust.edu.pk (M.T.M.); tayyaba.noor@scme.nust.edu.pk (T.N.); ayesharazi5030@gmail.com (A.R.); umer.adeel@scme.nust.edu.pk (M.A.U.); 2 School of Interdisciplinary Engineering and Sciences (SINES), National University of Science and Technology (NUST), H-12 Sector, Islamabad 44000, Pakistan; 3 Department of Applied Chemistry, Kyungpook National University, 80 Daehak-ro, Buk-gu, Daegu 41566, Republic of Korea; muzammil.nibge.pk@gmail.com

**Keywords:** dye sensing, methyl orange, adsorption kinetics, 2D-MoS_2_@ZIF-67 nanocomposite, MoS_2_ nanosheets, water purification

## Abstract

The zeolitic imidazolate framework-67 (ZIF-67) adsorbent and its composites are known to effectively remove organic dyes from aqueous environments. Here, we report a unique crystalline MoS_2_@ZIF-67 nanocomposite adsorbent for the efficient removal of methyl orange (MO) dye from an aqueous medium. In situ synthetic techniques were used to fabricate a well-crystalline MoS_2_@ZIF-67 nanocomposite, which was then discovered to be a superior adsorbent to its constituents. The successful synthesis of the nanocomposite was confirmed using XRD, EDX, FTIR, and SEM. The MoS_2_@ZIF-67 nanocomposite exhibited faster adsorption kinetics and higher dye removal efficiency compared with its constituents. The adsorption kinetic data matched well with the pseudo-second-order model, which signifies that the MO adsorption on the nanocomposite is a chemically driven process. The Langmuir model successfully illustrated the MO dye adsorption on the nanocomposite through comparing the real data with adsorption isotherm models. However, it appears that the Freundlich adsorption isotherm model was also in competition with the Langmuir model. According to the acquired thermodynamics parameters, the adsorption of MO on the MoS_2_@ZIF-67 nanocomposite surface was determined to be spontaneous and exothermic. The findings of this research open an avenue for using the MoS_2_@ZIF-67 nanocomposite to efficiently remove organic dyes from wastewater efflux.

## 1. Introduction

Around the world [1,2], freshwater reservoirs are polluted by various pollutants, e.g., oils, heavy metal ions, and organic wastes (i.e., dyes) [3]. Dyes have been successfully utilized in various industries, such as the food, paint, textile, paper, pigment, and cosmetics industries. Currently, around 100,000 types of dyes are commercially produced. Around 1.6 million tons of various dyes are utilized annually, and about 10–15% of dyes are wasted [4]. Organic dyes are resistant and tolerant to aerobic ingestion and oxidizing media [5] due to the presence of various benzene rings in organic dyes. Statistically, around 20% of the production of dyes is wasted in industrial sewage [6,7]. The dissipation of colourful effluents into aqueous media obstructs sunlight and consequently hampers the process of photosynthesis in aquatic plants and algae [8]. Moreover, organic dyes have been proven to adversely affect the metabolites of human beings and animals because of their mutagenicity, toxicity, and carcinogenicity [9]. 

To fix the crucial challenge of water impurity, various plans of action have been scrutinized. Coagulation, adsorption, flocculation, ion exchange, biodegradation, and oxidation are the main approaches that have been evaluated in this regard [10,11,12,13,14,15,16,17]; however, each method has merits and demerits. Among these techniques, adsorption has been greatly used because of its efficiency, easy functioning, diversity, low energy consumption, and cost-efficiency [11,12]. Among the traditional adsorbents, mesoporous silica, activated carbon, and polymer microspheres are widely used in adsorption applications [10,13]. Activated carbon is used commonly because of its large surface area and increased adsorption magnitude; however, its complex regeneration process and high cost obstruct its application severely [14,15]. Therefore, it is important to develop inexpensive, readily available, and durable adsorbents for rapid, efficient dye removal in large quantities. Zeolites and nanocomposites outperform activated carbon in their role as affordable adsorbent substances due to their porosity in nanometres, crystalline structure, ion-exchange capability, high surface area with respect to volume, efficacy, and, particularly, their accessibility in large reusable deposits at a comparatively low price [16,17].

The two-dimensional MoS_2_ nanosheet, a graphene analogue, is representative of two-dimensional transition metal dichalcogenides (TMDCs). A single Mo atom is covalently bonded to two layers of S atoms, forming the layered structure S-Mo-S. They have strong in-plane and weak out-of-plane bonding [18]. Recently, MoS_2_ gained significant attention because of its various unique properties, such as its large surface area [19] and the presence of a large number of active sites [10,20], along with enhanced optical, piezoelectric, electronic, and photocatalytic properties [21,22]. Through controlling the synthetic strategies for 2D MoS_2_ nanosheets, the specific morphology and number of layers can be tuned to reveal more active sites for the adsorption process. Fang et al. synthesized a flower-like sponge structure of MoS_2_ via the hydrothermal method with an adsorption capacity of 127.39 mg/g for Rhodamine B [20]. Song et al. reported MoS_2_ nanosheets with an adsorption capacity of 81.25 mg/g for RhB [19]. Qiao et al. illustrated the hierarchical structure of MoS_2_ for methylene blue adsorption [23]. Chao et al. reported the adsorption of doxycycline antibiotics by layered MoS_2_ nanofilm [24]. Qiao et al. also reported that ultrathin sheets of MoS_2_ could be efficiently used for the adsorption of methylene blue from aqueous media [25]. They demonstrated extraordinary adsorption, particularly of cationic dyes, namely MO, MB (methylene blue), CR (Congo red), etc. [19,26]. Magnetic Fe_3_O_4_-nanoparticle-enriched MoS_2_ nanosheets were used for CR adsorption, and because of their magnetic properties, they could be easily separated from suspension [27].

When MoS_2_ is introduced in aqueous media for dye adsorption, [MoO_4_]^−2^ ions are released and form complexes with cationic dye [(Dye^+^)_x_ + ([MoO_4_]^−2^)_y_], followed by an exhibition of prompt precipitation. However, anionic dyes repel [MoO_4_]^−2^ ions and consequently reduce their removal capacity [26]. Despite the above-mentioned excellence of MoS_2_ in dye removal from water, the release of molybdate ions [(MoO_4_)^−2^] from MoS_2_ upon dissolution is considered a secondary source of pollution [28].

However, zeolite imidazole frameworks (ZIFs), a class of metal–organic frameworks (MOFs), have been investigated for water treatment applications with excellent adsorption efficiencies [29]. ZIF-67 is iso-structural to ZIF-8 and is known for its large surface area, less density, adjustable pore size, and outstanding flexibility [30]. ZIF-67, a highly crystalline and porous MOF, contains a bridge between anions of methyl-2-imidazole and cations of cobalt metal [31]. Various research groups have reported superior adsorption of ZIF-67 toward several dyes [29,32,33].

In this work, a new composition of MoS_2_ with ZIF-67 is reported for dye adsorption from an aqueous medium. Methyl orange (MO) dye was selected as a test dye for this study. ZIF-67 has been reported to show excellent absorption properties due to its porous structure; MoS_2_ nanosheets’ large surface area also contributes to dye adsorption. In this study, MoS_2_ was incorporated with ZIF-67 at different concentrations to develop high-surface-area porous composites enriched with heteroatoms. 

The objective of the current study was to analyse the adsorption isotherms, adsorption kinetic models, the effect of pH, and the effect of temperature on the adsorption ability of MoS_2_@ZIF-67 nanocomposite for MO. To the best of our knowledge, this composite has not been reported before for methyl orange adsorption from an aqueous medium. 

## 2. Materials and Methods

All required chemicals and materials utilized for the synthesis of MoS_2_@ZIF-67 composite, MoS_2_ (2D) nanosheets, and ZIF-67 were of analytical grade and used without any additional purification step. Chemicals used in the whole experimentation are 2-Methylimidazol, Co(NO_3_)_2_·6H_2_O (>99% purity), bulk MoS_2_ powder (particle size less than 2 µm) (>99% purity), potassium nitrate (KNO_3_) (Sigma Aldrich, Darmstadt, Germany), ethanol (95% purity), conc. HCl (Merck, Darmstadt, Germany) (37% conc.) and methyl orange (Fischer Scientific Company, Waltham, MA, USA). A locally purchased deionized water was used throughout the experimentation.

### 2.1. Exfoliation of MoS_2_ to Prepare 2D MoS_2_ Nanosheets

First, 4.0 g potassium nitrate (KNO_3_) was added in 10.2 mL concentrated HCl in a round-bottom flask. This solution was then shaken to ensure a thorough blending before 0.8 g MoS_2_ bulk powder was added. The mixture was ultra-sonicated for about one hour at a 35–45 °C temperature range (with toxic gases collection setup). After sonication, the reaction was quenched in an ice bath for about 7 min. Then, the reaction mixture was centrifuged at 1000 rpm for 30 min to separate nanosheets of MoS_2_ from its bulk powder, and the supernatant fraction was again centrifuged at a higher rpm (8000) for 30 min, and the precipitated product was collected. The collected product was then dried at 70 °C in the air [34].

### 2.2. Synthesis of ZIF-67

ZIF-67 was synthesized through following a previously reported protocol [35]. A total of 0.5 g (1.717 mmol) Co(NO_3_)_2_·6H_2_O was mixed in 25 mL ethanol followed by the dropwise addition of 1 g (12.18 mmol) 2-Methylimidazol solution, made in 25 mL ethanol separately. Afterward, the mixture was stirred for 24 h at room temperature and centrifuged for 15 min at 4000 rpm for product collection. The product was washed multiple times with ethanol and then dried at 80 °C in a vacuum oven overnight.

### 2.3. In Situ Synthesis of Composite

**Figure 1:**
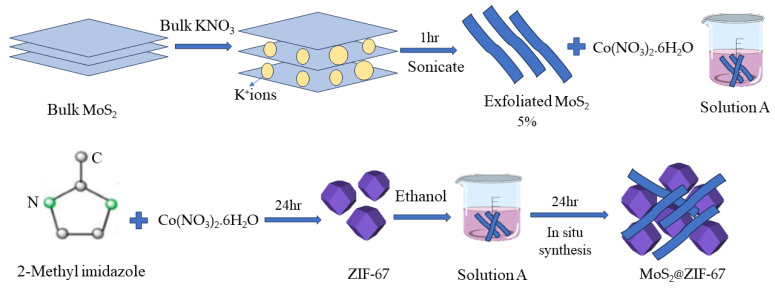
Schematic representation of the exfoliation MoS_2_ to prepare 2D MoS_2_ nanosheets and preparation solution A for in situ synthesis of MoS_2_@ZIF-67 nanocomposite through mixing with prepared ZIF-67 solution.

For the synthesis of the composite, 0.026 g MoS_2_ (2D) nanosheets (obtained via the chemical exfoliation method in Section 2.1) were added in 25 mL ethanol. The suspension was sonicated for 15 min, and 0.495 g (1.7 mmol) Co(NO_3_)_2_·6H_2_O was added to the suspension and stirred for 3 h (Solution A). Next, 1 g (12.18 mmol) of 2-methyl imidazole was added in 25 mL ethanol and stirred for 25 min (Solution B). Solution B was added dropwise into solution A and stirring continued for 24 h at room temperature. The product was then collected via centrifugation at 4500 rpm for 15 min, washed 3 times with ethanol, and dried at 80 °C in a vacuum oven overnight. Figure 1 shows a schematic representation of the steps taken to synthesize the MoS_2_@ZIF-67 nanocomposite. The MoS_2_ (2D, nanosheet) content of 5% by mass was selected based on previously reported work [36]. The synthesized nanocomposite was later pronounced as MoS_2_@ZIF-67, with exfoliated MoS_2_ (2D nanosheets) accounting for 5% by mass. To the best of our knowledge, this study is the first to report the technique employed for the in-situ production of MoS_2_@ZIF-67 nanocomposite.

### 2.4. Adsorption Experimentation

To interpret the adsorption isotherm and kinetic models, a 25 ppm stock solution of MO was prepared. The stock solution was then diluted to obtain 5, 10, 15, and 20 ppm concentrations for the calibration curve. This was performed to calibrate absorbance as a function of MO concentration and to avoid errors associated with changes in epsilon with temperature (for the Beer–Lambert’s law for UV-Vis measurements to determine other unknown concentrations). All experimentation was conducted through adding 0.075 g of adsorbent material in a 50 mL adsorptive volume of MO solution with continuous stirring at 400 rpm. After every 5 min until 90 min, a 5 mL sample was taken from the mixture, and the adsorbent material was separated via centrifugation at 4000 rpm. The dye concentration in the solution was evaluated using a UV-Vis spectrophotometer at λ_max_ of MO (λ_max_ = 465 nm). The adsorption capacities at equilibrium for different time intervals were calculated using dye concentrations at different time intervals. This was performed to evaluate the efficiencies of different adsorbents. To study the effect of pH, the desired pH of the solution was maintained using 0.1 M NaOH and 1 M HCl solution.

### 2.5. Characterization

An X-ray diffractometer (STOE, working at 40 kV voltage and 40 mA current) was used to verify phase purity and the crystal structure of the samples. The fine powder of synthesized samples was scanned in 2θ range of 5–80 degrees, whereas scanning electron microscopy (SEM, JEOL JSM-64900, JEOL Ltd., Tokyo 196-8558, Japan) was used to analyse the particle size of the samples and morphology. A Fourier-transform infrared spectrometer (FTIR, PerkinElmer, SpectrumTM100, Shelton, CT 06484-4794, USA) was employed to analyse IR spectrum data within the 400–4000 cm^−1^ scan range. An energy-dispersive X-ray spectrometer (EDX) (51-ADD0007, Oxford Instruments, Wiesbaden, Germany) was used to confirm the homogenous distribution of elements in the nanocomposite. A UV-Vis spectrophotometer (SPECORD 200 PLUS, analytikjena, Jena, Germany) was used for the adsorption studies. The details behind the adsorption studies will be discussed in Section 3. 

## 3. Results

**Figure 2:**
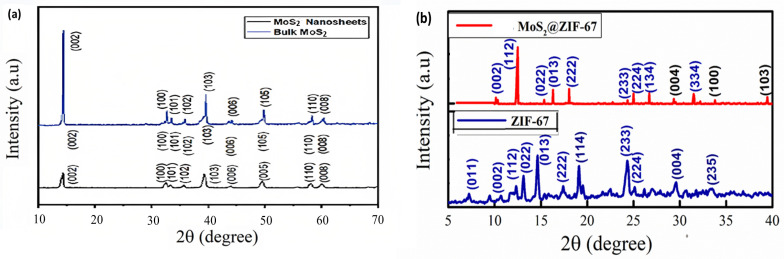
(**a**) XRD patterns for bulk MoS_2_ and its 2D nanosheets; (**b**) XRD spectra of pure ZIF-67 and 2D MoS_2_@ZIF-67 nanocomposite.

Figure 2a shows the XRD data plots for bulk MoS_2_ powder and 2D MoS_2_ nanosheets. The diffraction peaks detected at 29.46°, 32.30°, 33.72°, 38.52°, 44.12°, 46.51°, 54.50°, and 69.24° correspond to the (004), (100), (101), (103), (006), (105), (106), and (201) planes, respectively, which is in perfect agreement with the diffractogram for MoS_2_ hexagonal phase (JCPDS card No. 37-1492) [37]. The exfoliated 2D MoS_2_ nanosheets were found to possess substantially lower intensities in the diffractogram than the bulk MoS_2_ material. We would like to mention that MoS_2_@ZIF-67 composites were prepared through an in situ, co-precipitation technique which involved a suspension of exfoliated MoS_2_ nanosheets during ZIF-67 synthesis. It changed the nucleation of ZIF-67 from homogeneous to heterogeneous, considering the growth onto the surface of the nanosheets with the precipitation of ZIF-67. We speculate that this mechanism of nucleation or precipitation can retard the uniform growth of a nanocomposite and can lead to preferential growth along certain directions, thereby altering the intensity ratios of some planes when subjected to XRD characterization.

Figure 2b shows the diffraction peaks for ZIF-67 and MoS_2_@ZIF-67. For ZIF-67, the diffraction peaks were detected at 2θ values of 7.31°, 10.36°, 12.72°, 14.40°, 16.45°, 18.04°, 22.15°, 24.53°, 25.62°, 26.70°, 29.67°, 30.62°, and 32.43°, which correspond to the (011), (002), (112), (022), (013), (222), (114), (233), (224), (134), (044), (334), and (235) planes, respectively. Our XRD results were in agreement with the previously reported diffractograms of ZIF-67 [29]. The XRD plot for the MoS_2_@ZIF-67 nanocomposite was mostly following pure ZIF-67 because of its higher percentage. The sharp and dominant peaks in the XRD diffractogram for the nanocomposite indicated that ZIF-67 was highly crystalline and perfectly maintained its crystallinity during the synthesis of the nanocomposite. Peaks with small intensities were observed at 29.35°, 33.56°, and 39.43°, corresponding to the plane values of (004), (100), and (103), respectively. These planes were indexed to 2D MoS_2_ nanosheets, which indicate the presence of MoS_2_ nanosheets in the nanocomposite. 

**Figure 3:**
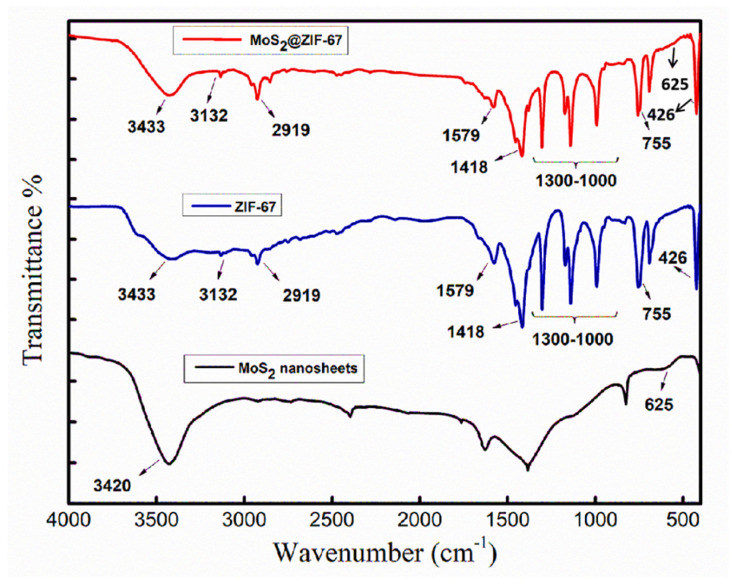
FTIR spectrum for the synthesized samples of ZIF-67, 2D MoS_2_ nanosheets, and MoS_2_@ZIF-67 nanocomposite.

Fourier-transform infrared (FTIR) analysis was used to confirm the bonds and chemical composition of the synthesized samples (Figure 3). The Mo-S stretching peak appeared at 625 cm^−1^ in the FTIR spectrum of the MoS_2_@ZIF-67 nanocomposite [38,39], while the peak around 3400 cm^−1^ is assigned to O-H stretching vibrations. The same spectrum also showed stretching vibrations at 1418 and 1579 cm^−1^ for the imidazole ring. The peaks in the region of 600 cm^−1^ to 1500 cm^−1^ were because of the bending and stretching vibrational modes of the imidazole batch. The peaks at 755 cm^−1^ and 1000–1300 cm^−1^ exhibited the out-of-plane and in-plane bending vibrations of the imidazole group, respectively. The peak at 2919 cm^−1^ indicated the stretching vibrations of the C-H bond from the non-aromatic (aliphatic) methyl group. The stretching vibrations of the aromatic ring in the imidazole group showed their peak at 3132 cm^−1^. The FTIR spectrum for pure ZIF-67 and 2D MoS_2_@ZIF-67 nanocomposite showed almost the same peaks because MoS_2_ was difficult to observe via FTIR analysis with only 5% of MoS_2_ content in the final composite. The obtained FTIR data matched well with the literature [40,41].

**Figure 4:**
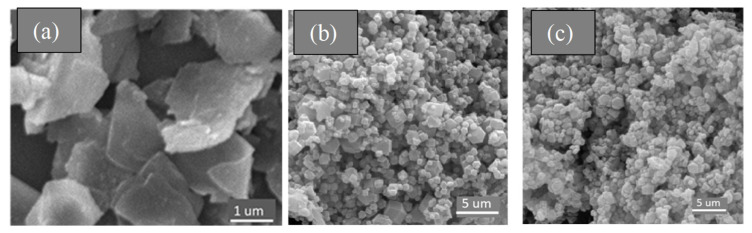
SEM micrographs: (**a**) exfoliated MoS_2_ nanosheets, (**b**) ZIF-67, and (**c**) MoS_2_@ZIF-67 nanocomposite.

**Figure 5:**
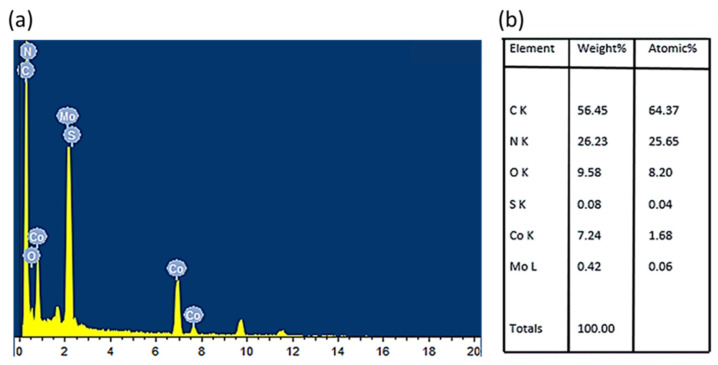
(**a**) EDX spectrum and (**b**) elemental composition percentage of MoS_2_@ZIF-67 nanocomposite.

Figure 4 represents the SEM images of the synthesized samples. Figure 4a shows the SEM images for exfoliated MoS_2_ (nanosheets). Figure 4b shows the SEM image for nearly pure ZIF-67 with rhombic dodecahedron morphology. The SEM micrograph of the synthesized MoS_2_@ZIF-67 nanocomposite was found to be similar to ZIF-67 (see Figure 4c in comparison with Figure 4b). We speculate that very low MoS_2_ content (by mass percentage) is the main reason behind little or no morphological difference between ZIF-67 and MoS_2_@ZIF-67 nanocomposite. Low MoS_2_ content in the ultimate nanocomposite was confirmed via EDX analysis (shown in Figure 5). The elemental analysis of the nanocomposite revealed the weight percentages of Mo and S to be 0.42 and 0.08, respectively. Such a little portion of MoS_2_ nanosheets may not render any significant change in the morphology of the nanocomposite. Higher percentages of Co around 7.24 can also be recorded in EDX analysis.

### 3.1. Adsorption Kinetics

The adsorption kinetics is based on the analysis of time-dependent variables influencing the reaction rates. It also assists to evaluate the adsorption capacity of adsorbent material as well as the reaction mechanism and mass transfer. 

**Figure 6:**
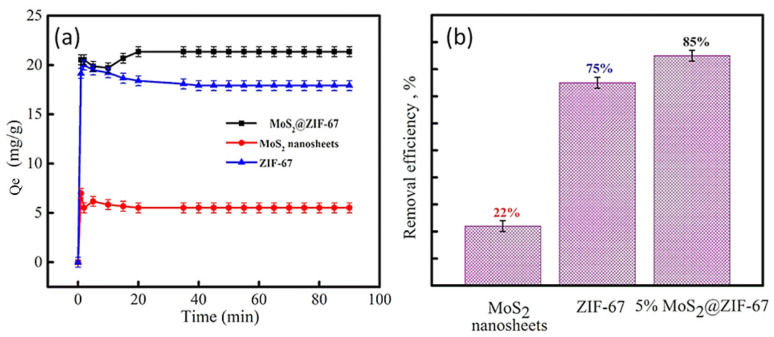
(**a**) Variation in adsorption capacity of various adsorbents towards MO dye as a function of time; (**b**) MO removal efficiency for 2D MoS_2_ nanosheets, ZIF-67, and MoS_2_@ZIF-67 nanocomposite.

Figure 6a shows the adsorption capacity of different adsorbents for MO adsorption from an aqueous medium. It is evident from the plot that the MoS_2_@ZIF-67 nanocomposite had the highest adsorption capacity compared to ZIF-67 and MoS_2_ (2D nanosheets). Since an equilibrium level of adsorption capacity was achieved after some time (in every sample), we used the symbol *Q_e_* to identify the adsorption capacity here. The adsorption capacities were used to calculate the MO removal efficiency (Figure 6b). The dye removal efficiency for the nanocomposite was found to be 85%, while it was 75% and 22% for ZIF-67 and MoS_2_ nanosheets, respectively. The effect of pH on adsorption properties has been investigated in Section 3.3, where pH 5 was determined to be the optimal value. Therefore, all the adsorption studies were performed at this pH. 

To get to the bottom of the adsorption process, the two most frequently used kinetic models known as pseudo-first-order and pseudo-second-order models were investigated for MO adsorption on the surface of MoS_2_@ZIF-67 nanocomposite. The same models were also applied to 2D MoS_2_ nanosheets and ZIF-67 adsorbents for comparison with the nanocomposite. 

According to the pseudo-first-order model, the extent of the adsorption rate is associated with the number of active sites on the adsorbent’s surface (i.e., the sites available for MO adsorption, in this case). The mathematical expression for the pseudo-first-order kinetic model is represented using the following equation (Equation (1)) [42]:

(1) $$ln\left(Q_{e}-Q_{t}\right)=lnQ_{e}-K_{1}t$$

where *K*_1_ is the rate constant of the pseudo-first-order kinetic model for the adsorption process (min^−1^). *Q_e_* is the equilibrium adsorption capacity of the adsorbent. *Q_t_* is the amount of MO dye adsorbed on the adsorbent’s surface at time ‘*t*’ (in minutes). In other words, *Q_t_* is the adsorption capacity of the adsorbent at time “*t*” (in min). 

The second-order model enlightens the adsorption data over the total adsorption time. The following equation serves as the mathematical expression for the pseudo-second-order model (Equation (2)) [43]:

(2) $$\frac{t}{Q_{t}}=\frac{1}{K_{2}Q_{e}^{2}}+\frac{t}{Q_{e}}$$

where *K*_2_ is the rate constant of the pseudo-second-order kinetic model for the adsorption process (min^−1^).

The assumption behind the pseudo-first-order model is that physisorption regulates the rate at which adsorbate molecules adhere to the adsorbent’s surface. On the other hand, the pseudo-second-order model assumes that the adsorption process is limited by chemisorption. Thus, if the adsorption data reasonably correlate with both of these two models, there is a physiochemical alliance between the adsorbent and the adsorbate [44].

**Figure 7:**
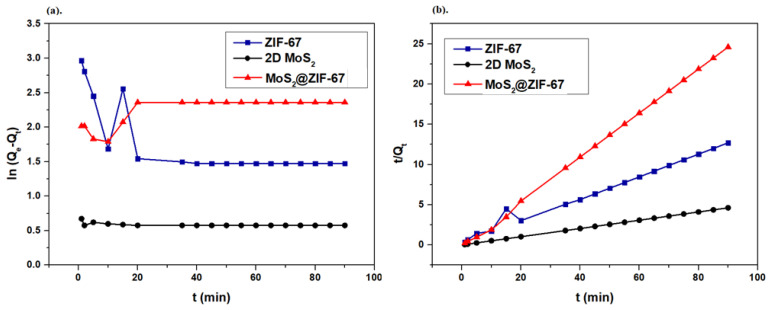
(**a**) The pseudo-first-order kinetic model and (**b**) the pseudo-second-order kinetic model for methyl orange adsorption by ZIF-67, 2D MoS_2_ nanosheets, and MoS_2_@ZIF-67 nanocomposites.

The actual data plots (shown in Figure 7a,b) were matched with the abovementioned kinetic models. The measure of competency between the data plots and the above two kinetic models could be examined using the correlation coefficient (R^2^). The kinetic parameters for these models after correlating with the actual data plots are listed in Table 1. The value of the correlation coefficient (R^2^) for the pseudo-second-order model when applied to ZIF-67, 2D MoS_2_ nanosheets, and MoS_2_@ZIF-67 nanocomposite is greater than 0.98. Contrarily, the values of R^2^ for the pseudo-first-order kinetic model are 0.3193, 0.5657, and 0.5893 for MoS_2_ nanosheets, ZIF-67, and MoS_2_@ZIF-67, respectively. This illustrates that the adsorption kinetics is supported by the pseudo-second-order model and, therefore, it is most likely that chemisorption controls the adsorption of MO on the synthesized nanocomposite and its components. Thus, we speculate that the addition of polar MoS_2_ (nanosheets) into the ZIF-67 matrix tends to enhance attractive dipole interactions between the polar MO dye and the nanocomposite. Therefore, one may conclude that the MoS_2_@ZIF-67 nanocomposite may only be suitable for the removal of polar dyes from aqueous media. However, this needs to be further investigated in future studies.

### 3.2. Adsorption Isotherms

Two adsorption isotherms named the Langmuir adsorption isotherm and Freundlich adsorption isotherm were used to analyse the process of dye adsorption. Such studies are usually carried out to investigate whether adsorption is a monolayer or multilayer process. It is important to briefly explain these two adsorption isotherm models before comparing them with our data plots.

#### 3.2.1. Langmuir Adsorption Isotherm

The Langmuir adsorption isotherm can be expressed using the following mathematical expression (Equation (3)):

(3) $$\frac{C_{e}}{Q_{e}}=\frac{1}{Q_{m}K_{L}}+\frac{C_{e}}{Q_{m}}$$

where *Q_e_* is the extent of adsorption (or the adsorption capacity) at equilibrium (in mg/g), *C_e_* is the concentration of dye left in aqueous solution at equilibrium (in mg/L), *K_L_* is the coefficient of Langmuir adsorption, and *Q_m_* is the extent of monolayer adsorption of the adsorbate on the adsorbent’s surface (in mg/g). The slope and intercept obtained from the graph of *C_e_*/*Q_e_* versus *C_e_* gives the values of *Q_m_* and *K_L_*, respectively. *R_L_* is a dimensionless factor exhibited by the following equation (Equation (4)). It gives information about the compatibility of the adsorption process with the given adsorption system.

(4) $$R_{L}=\frac{1}{1+C_{0}K_{L}}$$

The adsorption process would be favourable if *R_L_* < 1, and unfavourable when *R_L_* > 1. *R_L_* = 1 represents a linear process and *R_L_* = 0 is the indication of an irreversible process. This model assumes that the adsorption is governed by the development of the monolayer on the homogenous surface of the adsorbent [45].

#### 3.2.2. Freundlich Adsorption Isotherm

The equation for the linear Freundlich isotherm is given in Equation (5):

(5) $$lnQ_{e}=lnK_{f}+\left(\frac{1}{n}\right)lnC_{e}$$

where *K_f_* is Freundlich constant to narrate the adsorption capacity at equilibrium (i.e., *Q_e_* in mg/g) and n describes the suitable adsorption system. This model assumes that adsorption is based on the development of a multilayer on a heterogeneous surface [46]. 

#### 3.2.3. The Experimental Data Plots vs. the Adsorption Isotherm Models

**Figure 8:**
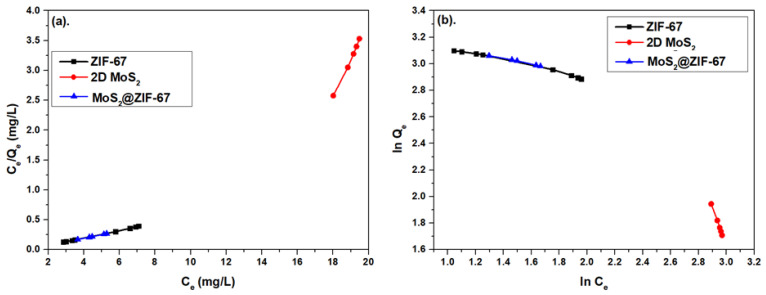
Comparison plots for (**a**) Langmuir and (**b**) Freundlich adsorption isotherms for methyl orange adsorption by ZIF-67, MoS_2_ nanosheets, and MoS_2_@ZIF-67 nanocomposite.

Figure 8a shows the plots of *C_e_*/*Q_e_* vs. *C_e_* for ZIF-67, 2D MoS_2_ nanosheets, and MoS_2_@ZIF-67 nanocomposite. The *K_L_*, *Q_m_*, *R_L_*, and R^2^ values from these data plots are listed in Table 2. R^2^ values for all three different adsorbents are greater than 0.99, which is the manifestation of a good match between our experimental data with the Langmuir adsorption isotherm model. Therefore, monolayer adsorption is most likely to take place on the surface of all three different adsorbents. *R_L_* is far less than one for each adsorbent, though its numerical value is lowest for the MoS_2_@ZIF-67 nanocomposite. Thus, monolayer adsorption is expected to be most significant on the surface of the nanocomposite. 

Figure 8b shows the plot between ln *Q_e_* versus ln *C_e_* for the Freundlich adsorption isotherm (when applied to all three different adsorbents). Numerical data of *n* and *K_f_* were employed to understand the extent of the favourability of the multilayer adsorption process. The adsorption process would be favourable if the value of 1/*n* lies between zero and one [46]. The values of *K_f_*, 1/*n*, and R^2^ for the Freundlich adsorption isotherm model are also listed in Table 2. For pure 2D MoS_2_ nanosheets, 1/n is greater than three. However, its values are less than one for ZIF-67 and the MoS_2_@ZIF-67 nanocomposite adsorbents. Secondly, R^2^ values are greater than 0.99 but less than 1 for all three different adsorbents. Based on the values of 1/*n* and R^2^, it can be stated that the Freundlich adsorption isotherm model also matches very well the ZIF-67 and MoS_2_@ZIF-67 samples, respectively.

When comparing the parameters listed in Table 2 (especially R^2^, *R_L_*, and 1/*n*), we speculate that significant competition existed between the monolayer and multilayer adsorption when it comes to the synthesized MoS_2_@ZIF-67 nanocomposite. The same might be true for the ZIF-67 sample; however, multilayer adsorption of MO on the surface of 2D MoS_2_ is not feasible because 1/*n* for this sample is greater than one. *R_L_* for the Langmuir adsorption isotherm is less than one for each adsorbent. The value of *R_L_* for ZIF-67 is closer to zero, while that for 2D MoS_2_ is much higher. Thus, 2D MoS_2_ may be contributing to monolayer adsorption. 

### 3.3. Effect of pH

**Figure 9:**
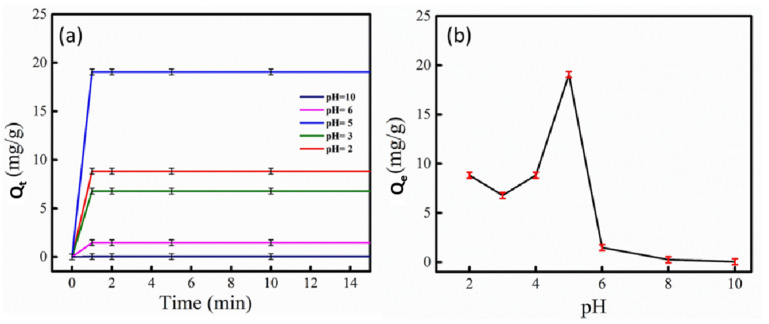
Effect of pH on methyl orange adsorption by MoS_2_@ZIF-67 nanocomposite (**a**) Effect of pH on the extent of adsorption for the nanocomposite (Q_t_) (**b**) Equilibrium adsorption capacity (Q_e_) of the nanocomposite.

The pH of the solution has a prime role in the adsorption process because it influences the adsorbent’s surface charge and affects the ionization process of adsorbate molecules [47]. Figure 9a displays the effect of pH on the extent of adsorption for the nanocomposite (Q_t_). Thus, the adsorbed amount of MO on the surface of MoS_2_@ZIF-67 nanocomposite promptly increased in the first 10 s, and then equilibrated nearly after 1 min. Figure 9b shows the plot of the equilibrium adsorption capacity versus the pH of the solution. The adsorption of MO on the MoS_2_@ZIF-67 nanocomposite was favourable in an acidic medium, and the highest removal was noted at a pH value of five. Significant reduction in MO adsorption was spotted in the 6–10 and 2–4 pH ranges. The lowest adsorption capacity was recorded at pH 10. Therefore, the optimal pH value for the adsorption of MO on the surface of MoS_2_@ZIF-67 nanocomposite was found to be five. 

This behaviour of the nanocomposite could be explained based on the observations reported by Hu et al. [29]. More than 95% of this nanocomposite is a ZIF-67 metal-organic framework. In acidic and neutral solutions, the charge on the ZIF-67 surface becomes positive, which reduces to zero at a pH of 8.7. Above 8.7, the ZIF-67 surface becomes completely negative and the equilibrium adsorption capacity (*Q_e_*) decreases significantly due to the electrostatic repulsion. Although л-л stacking is functional between the benzene ring of the dye and the adsorbent molecule, the electrostatic interaction was presumably the main source of adsorption [47].

### 3.4. Temperature Effect

**Figure 10:**
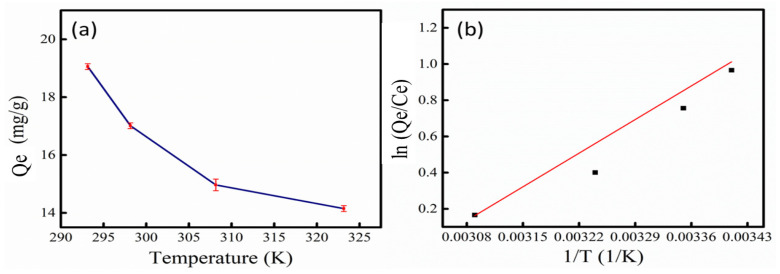
(**a**) Decrease in methyl orange adsorption by MoS_2_@ZIF-67 nanocomposite with increasing temperature; (**b**) Adsorption distribution plot between 1/T and ln (*Q_e_*/*C_e_*).

Temperature is one of the most dominant factors in the adsorption process. A significant temperature rise enhances the diffusion rate of the adsorbate and reduces the solution viscosity, which may influence the adsorbent’s adsorption capacity at equilibrium [48,49]. Therefore, it is important to investigate the effect of temperature on MO adsorption on the MoS_2_@ZIF-67 nanocomposite. The results showed a decreasing trend in the equilibrium adsorption capacity of the MoS_2_@ZIF-67 nanocomposite from 19.0 mg/g to 14.11 mg/g through increasing the temperature from 290 K to 330 K, as exhibited in Figure 10a. The remarkable decrease in adsorption capacity suggests that MO adsorption is not feasible at high temperatures for MoS_2_@ZIF-67 nanocomposites. Therefore, the MO adsorption on the MoS_2_@ZIF-67 nanocomposite is directly related to the solution temperature. The acquired results nearly followed the same trends as for MO adsorption on MoS_2_@PDOPA, which were previously reported by Q. Huang et al. [50].

Thermodynamic studies were also performed, which provided supplementary information on intrinsic energy changes associated with the adsorption process. For a proper understanding of the thermodynamics of the adsorption process, three thermodynamic parameters were studied, i.e., enthalpy change (Δ*H*^0^), entropy change (Δ*S*^0^), and change in Gibbs free energy (Δ*G*^0^) (Table 3). The following equations were used to understand the thermodynamics of the adsorption process:

(6) $$\Delta G^{0}=-RTlnK_{\alpha }$$

(7) $${∆G}^{0}={∆H}^{0}-T{∆S}^{0}$$

Plugging Equation (6) into Equation (7) results in

(8) $$lnK_{\alpha }=\frac{\Delta S^{0}}{R}-\frac{\Delta H^{0}}{RT}$$

where

$$K_{\alpha }=\frac{Q_{e}}{C_{e}}$$

where

*K_α_* = adsorption distribution coefficient;
*Q_e_* = adsorption capacity of the (nanocomposite) adsorbent at equilibrium (mg/g);
*C_e_* = adsorbate concentration at equilibrium (mg/L);
*R* = universal gas constant (8.314 J/mol K);
*T* = temperature (K).

Δ*S*^0^ and Δ*H*^0^ values were calculated from the intercept and slope of the ln *K_α_* versus 1/*T* graph, (Figure 10b). At various temperatures, Δ*G*^0^ values were shown to be negative (Table 3). These results demonstrated that MO adsorption on MoS_2_@ZIF-67 nanocomposite was a spontaneous process. Additionally, the decrease of negative values from −2.840 kJ/mol to −0.713 kJ/mol through increasing the temperature from 290.15 K to 330.15 K indicated a reduction in driving force for the adsorption process at elevated temperatures [50]. The negative values of ${∆H}^{0}$ showed that the adsorption process was exothermic. MO adsorption on 2D MoS_2_@ZIF-67 nanocomposite’s surface was more suitable at lower temperatures as compared to the higher ones. MO adsorption was mainly controlled via the chemisorption phenomenon because all the obtained values of $\Delta H^{0}$ were in the range of 0–40 kJ/mol [50]. This result was following the above-mentioned interpretation. $\Delta S^{0}$ negative values indicate that the molecular motion of MO got restricted after adsorption on the surface of the nanocomposite. All thermodynamic parameters strongly recommended significant MO adsorption on MoS_2_@ZIF-67 nanocomposite. This nanocomposite could be used as an advanced material for the adsorption of MO dye for its efficient removal from aqueous media.

## 4. Conclusions

In this research work, we successfully synthesized a series of highly crystalline MoS_2_@ZIF-67 nanocomposites to be used as a versatile adsorbent material for MO removal from an aqueous medium. The synthesized nanocomposite turned out to be much more efficient in dye removal compared to its constituents. The X-ray diffraction (XRD) analysis of the MoS_2_@ZIF-67 nanocomposite predominantly exhibited the characteristic peaks of pure ZIF-67 due to its higher-percentage composition. However, additional peaks with lower intensities were observed at 29.35°, 33.56°, and 39.43°, corresponding to the crystal planes (004), (100), and (103), respectively. These planes were attributed to the presence of 2D MoS_2_ nanosheets within the nanocomposite. Although the weight percentage of Mo and S in the nanocomposite was found to be 0.42 and 0.08, respectively, the presence of such a small portion of MoS_2_ nanosheets did not significantly alter the morphology of the nanocomposite. Based on a comparison between the two kinetic models (i.e., pseudo-first order and second order), when applied to the adsorption of MO on the surface of the MoS_2_@ZIF-67 nanocomposite, the adsorption seemed to be purely governed by chemisorption. Similarly, when the adsorption isotherm models were applied to MO adsorption on the surface of the MoS_2_@ZIF-67 nanocomposite, we speculated that both the monolayer and multilayer adsorption processes are taking place in this system. Further studies, when performed on the nanocomposite, indicated that the temperature and pH of the MO solution significantly affected the adsorption capacity. Superior adsorption of MO on MoS_2_@ZIF-67 nanocomposite was achieved at room temperature at a pH value of five. According to the thermodynamic studies, the calculated value of Δ*G*^0^ indicated that the adsorption of MO was spontaneous and more efficient at lower temperatures. Regarding dye removal efficiency, the nanocomposite exhibited an impressive removal rate of 85%, while ZIF-67 and MoS_2_ nanosheets alone achieved removal rates of 75% and 22%, respectively. The application of the pseudo-second-order kinetic model resulted in correlation coefficients (R_2_) greater than 0.98 for ZIF-67, 2D MoS_2_ nanosheets, and the MoS_2_@ZIF-67 nanocomposite. Conversely, the R_2_ values for the pseudo-first-order kinetic model were 0.3193, 0.5657, and 0.5893 for MoS_2_ nanosheets, ZIF-67, and MoS_2_@ZIF-67, respectively. This indicates that the adsorption kinetics is best described using the pseudo-second-order model, suggesting that chemisorption processes predominantly control the adsorption of MO dye onto the synthesized nanocomposite and its constituents. The incorporation of polar MoS_2_ nanosheets into the ZIF-67 matrix likely enhances attractive dipole interactions between the polar MO dye and the nanocomposite, leading to improved dye removal efficiency. Consequently, it can be concluded that the MoS_2_@ZIF-67 nanocomposite shows promise for the effective removal of polar dyes from aqueous media. Thus, we recommend MoS_2_@ZIF-67 nanocomposite be further studied against different dyes to evaluate its suitability for the efficient treatment of wastewater from textile and other related industries.

## Figures and Tables

**Table 1:** Adsorption kinetics models’ parameters for methyl orange adsorption from aqueous media.

Dye	Adsorbent	*Q_e_* (mg/g)	Pseudo First Order Equation			Pseudo Second Order Equation		
Methyl orange		(Experimental)	*Q_e_* (Calculated) (mg/g)	*K*_1_ (L/min)	R^2^	*Q_e_* (mg/g)	*K*_2_	R^2^
	MoS_2_ nanosheets	5.51876	1.8325	0.0005	0.3193	19.49	6.982	1
	ZIF-67	17.9168	10.46	0.0005	0.5657	7.490	0.032	0.9854
	MoS_2_@ZIF-67 composite	21.34258	7.417	0.0003	0.5893	3.952	0.443	0.9994

**Table 2:** Adsorption isotherm parameters for various adsorbents.

Dye	Adsorbent	Langmuir Isotherms Constants				Freundlich Isotherms Constants		
Methyl orange		*K_L_* (L/mg)	*Q_m_* (mg/g)	R^2^	*R_L_*	*K_f_* (mg/g)	1/*n*	R^2^
	MoS_2_ nanosheets	0.0705	1.506	0.9961	0.3619	1.9440	3.057	0.9983
	ZIF-67	1.1077	15.673	0.999	0.0348	3.0982	0.2424	0.9939
	MoS_2_@ZIF-67 composite	1.2413	16.7504	0.9996	0.0312	3.060704	0.2183	0.9971

**Table 3:** Thermodynamic parameters for methyl orange adsorption by 5%MoS_2_@ZIF-67 nanocomposite.

Temperature (K)	∆*G*^0^ (KJ mol^−1^)	∆*H*^0^ (KJ mol^−1^)	∆*S*^0^ (KJ mol^−1^ K^−1^)
293.15	−2.84007	−2.66	−0.008
298.15	−1.87406	-	-
308.15	−1.02482	-	-
323.15	−0.71313	-	-

## Data Availability

The data that support the findings of this study are available from the corresponding author upon reasonable request.
